# Foliar dust and heavy metal deposit on leaves of urban trees in Budapest (Hungary)

**DOI:** 10.1007/s10653-020-00769-y

**Published:** 2020-11-13

**Authors:** Károly Hrotkó, Márta Gyeviki, Diószegi Magdolna Sütöriné, Lajos Magyar, Róbert Mészáros, Péter Honfi, Levente Kardos

**Affiliations:** 1 Department of Floriculture and Dendrology, Szent István University, Villányi Str. 35-43., Budapest, 1118 Hungary; 2 Department of Meteorology, Eötvös Loránd University, Pázmány Péter stny. 1/A, Budapest, 1117 Hungary; 3 Department of Soil Science and Water Management, Szent István University, Villányi Str. 35-43., Budapest, 1118 Hungary

**Keywords:** *Acer platanoides*, Air pollution, *Fraxinus excelsior*, Heavy metal content of leaves, *Tilia tomentosa*, Urban trees

## Abstract

This work considers dust deposition and the heavy metal (HM) content on leaves of urban trees (*Acer platanoides* L. ‘Globosum,’ *Fraxinus excelsior* L. ‘Westhof’s Glorie’ and *Tilia tomentosa* Moench.) in order to estimate the trees’ capacity to remove dust and HM from the air. Leaves were collected from the Buda Arboretum and from different streets of heavy traffic in Budapest, Hungary, during 2015 and 2016. At each site, five trees were sampled by collecting 6 leaves from each tree from the height of 2–3 m. Dust deposits on the leaves were removed by soaking the fresh foliage in distilled water for 20 h and then washed with ultrasound shaking. Afterward, the leaves were dried to constant weight and then they were digested in nitric acid–hydrogen peroxide treatment, and their Pb, Fe, Ni, Zn and Cu contents were measured using an inductively coupled plasma (ICP AS) spectrometer. The removed dust deposit was dried, and after a similar digestion treatment the Pb, Fe, Ni, Zn and Cu contents were measured using an AURORA AI 1200 AAS appliance. The HM deposit was calculated in mg m^–2^ leaf surface area. In 2015, the amount of foliar dust deposit from spring to autumn increased from 86.3 to 270.2 mg m^–2^. The most efficient tree species in trapping dust on their leaves was the silver linden (98.5–123.5 mg m^−2^), followed by the Norway maple (74.2–84.8 mg m^−2^) and the common ash (62.8–74.6 mg m^−2^). The deposit of HM elements showed seasonal differences: the quantity of Fe and Pb deposit on autumnal leaves increased five- to tenfold, while other heavy metals did not show accumulation. Silver linden with its pubescent (hairy) leaf surface proved to be most efficient in entrapping and retaining dust and heavy metals. The 60–100% higher Pb and Fe content of autumnal leaves indicate that over the season leaves may absorb Fe and Pb from the foliar dust. Our results confirmed that the foliar dust is a potential indicator for monitoring the HM content in the air. We also show that foliar dust deposits should be considered when estimating the capacity of urban trees to clean the air.

## Introduction

Atmospheric pollution causes serious human health problems in many urban communities; indeed, related effects such as discomfort and smog can also lead to economical and societal complications. Studies suggest that one of major sources of urban air pollution is due to traffic emitting CO_2_, CO, NO_x_, other gaseous compounds as well as dust and soot particulate matter (PM). Furthermore, this pollution causes significant environmental damages on vegetation, buildings and human health (Hosker and Lindbergh 1982; Davidson et al. 2005; Yang et al. 2005; Kampa and Castanas 2008; Apeagyei et al. 2010; Lu et al. 2010; Zupancic et al. 2015; Badamasi 2017).

Dust deposits on leaf of urban trees may contain particulate matter (PM), non-gaseous components, carbon compounds, metals, pollen and soil particles. Atmospheric particulate matter causes serious health problems, respiratory and vascular diseases all over the world (Davidson et al. 2005; Kampa and Castanas 2008). Metals are associated with higher road traffic in both urban and rural areas (Hosker and Lindbergh 1982; Apeagyei et al. 2010; Lu et al. 2010; Simon et al. 2011; Moreira et al. 2016; Badamasi 2017), and elevated levels of heavy metals (HM) in urban atmosphere are reported by Apeagyei et al. (2010) and Lu et al. (2010). Vehicles are major sources of HM (Pb, Zn, Cu, Ni and Fe) particles (Christoforidis and Stamatis 2009, Apeagyei et al. 2010; Lu et al. 2010; Moreira et al. 2016); thus, HM pollution under urban conditions is strongly associated with PM and traffic. The results of Apeagyei et al. (2010) and Lu et al. (2010) indicate that roadway dust may be important source of metals in runoff water and in atmospheric HM. The ten most important elements in atmospheric heavy metal pollution are in ranking: Fe, Al, Pb, Zn, Ti, Mn, Cu, V, Ni, Cr (Hoodaji et al. 2012).

Plants can be an important entrapping surface for dust principally in the cities (Chen et al. 2016). Plant leaves have been used as indicators and/or monitors of trace metal pollution (Jensen et al. 1992; Jim and Chen 2008; Petkovšek et al. 2008; Balasooriya et al. 2009; Sæbø et al. 2012; Simon et al. 2011, 2014; Badamasi 2017). Popović et al. (2010) and Tomašević et al. (2011) suggested the leaf Pb content for monitoring of atmospheric Pb pollution. Moreover, in some cases higher plants may give better quantifications for pollutant concentrations and atmospheric deposition than non-biological samples. Fine anthropogenic particles were often observed around and over the stomata which may affect the physiological characteristics of leaves (Sæbø et al. 2012; Simon et al. 2014). According to Tomašević and Aničić (2010), both fine (< 2 µm) and coarse (up to 50 µm) particles are responsible for increased leaf temperature and decreased light absorption; thus, they affect the photosynthesis of plants.

The removal of dust from the ambient air is one of the important environmental services of urban trees (Jensen et al. 1992; Jo and McPherson 1995; Yang et al. 2005; Jim and Chen 2008; Balasooriya et al. 2009; Sæbø et al. 2012; Simon et al. 2011, 2014; Chen et al. 2016). It is commonly accepted that urban trees play an important role in reducing air pollution due to their large leaf areas relative to other plants in green spaces (Jo and McPherson 1995; Yang et al. 2005; Jim and Chen 2008). Trees can remove large numbers of airborne particles, hence improving the quality of air in polluted environments (Jensen et al. 1992; Beckett et al. 1998; Chen et al. 2016). According to Beckett et al. (2000a), trees can successfully reduce the atmospheric level of fine, respirable particulates; therefore, they play a special role in effecting urban environment and human health. Beckett et al. (2000a) found that the finer, more complex structure of the foliage of the conifers *Pinus nigra* and × *Cupressocyparis leylandii* explained their much greater effectiveness at capturing particles. As Simon et al. (2011) reported, *Acer platanoides* and *Populus alba* could be considered as bioindicators for air pollution; however, they found no differences in the dust deposit on their leaves. Aksoy and Demirezen (2006) also suggested *Fraxinus excelsior* as a bioindicator of air pollution.

PM deposition depends on meteorological conditions (mainly precipitation and wind speed), on the physical characteristics of the particles (size and shape), on the morphological characteristics of the plants, canopy structure and the planting configuration (Beckett et al. 2000a; Yuan et al. 2009; Sæbø et al. 2012; Mori et al. 2015). The leaf structure, roughness of leaf surface, the presence of trichomes, stomata size and density may influence the dust and PM deposit on leaves (Tomašević and Aničić 2010; Simon et al. 2014; Mori et al. 2015). Rough leaf surfaces (Beckett et al. 2000b) and the presence of trichomas on *Eleagnus* × *ebbingei* (Mori 2015) are efficient in capturing PM. The highest trichoma density of *Celtis occidentalis* and *Acer negundo* leaves was linked with the highest dust deposition, while on the smoothest leaves of *Padus serotina* and *Quercus robur* smaller dust deposits were found (Simon et al. 2014). No significant connection was observed by Chen et al. (2016) between species leaf features and the PM capturing ability, so they conjecture further specific factors beyond leaf characteristics have greater influence on the PM deposition of plant surfaces. There are some seasonal differences in deposition of air pollutants on leaves of plants (Nowak et al. 2006; Mori et al. 2015) caused by weather conditions.

Urban trees represent the largest leaf area in parks and street plantations (Jo and McPherson 1995; Yang et al. 2005; Chen et al. 2016). The estimation of the air cleansing capacity of urban trees requires in situ measured data on various urban tree species concerning the dust and HM deposition on their leaves. However, the situation is more complicated as trees in street canyons may reduce the air circulation and therefore may lead to higher local PM concentrations. Further complicating matters, the impact of trees on air flow depends substantially on the surrounding built environment. Therefore, Chen et al. (2016) suggest designing proper configuration of planting spaces to improve the purification function of urban trees.

The evaluation of dust removal capacity of several common tree species requires investigations in interaction with urban conditions, but such measurements and data are very little known. In this work, we estimate the capacity of dust and HM removal of major urban trees in Budapest (Hrotkó et al. 2014a). In alleys and urban green spaces, the common ash (*Fraxinus excelsior* L.), the Norway maple (*Acer platanoides* L.) and the silver linden (*Tilia tomentosa* Moench) are widely planted in Budapest (Szaller et al. 2014). As little data are known about dust and HM entrapping capacity of common ash and Norway maple (Aksoy and Demirezen 2006; Sæbø et al. 2012) and no data on silver linden, our research aimed to estimate the dust and HM entrapping capacity of the major urban trees in Budapest under different air pollution conditions. During our investigation, our main goal was to quantify the dust and major HM deposition on leaves collected from commonly planted urban trees. Based on the literature, our hypotheses were: (1) the different leaf characteristics of investigated tree species influence the foliar dust entrapping capacity of leaves; (2) the different leaf characteristics of tree species influence the HM deposit on leaves of urban trees; (3) besides the Pb content of leaves, the detection of other HM elements might be suitable indicators of HM air pollution.

## Materials and methods

### Study area and tree species

The study was undertaken within Budapest, the capital of Hungary with a population of about 2 million inhabitants. The city is located on both side of the Danube River: between the dry continental Hungarian Plain and the more humid climate of Buda Hills. The annual mean temperature is 11.3 °C, total sunshine is 2079 h, and the annual rainfall (mean of 50 years) is about 550 mm, falling mainly in May or June and in the autumn, with large temporal variability each year. The city center areas are densely populated with busy roads, train tracks and urban green spaces. We chose four urban sites on both side of the Danube (Buda and Pest) from which three areas have heavy traffic (Krisztina Street, Andrássy Avenue and Karolina Street), while the fourth, the Buda Arboretum, is located away from heavy traffic charged roads. These sampling sites allow the comparison of each tree species within low and heavy traffic conditions. As our sampling sites were within 2 km of reference stations for the Hungarian Air Quality Network (HAQN) (see Table 1), we were able to use their data on air PM10 pollution. The selected tree species are deciduous urban trees commonly planted in East Central European cities, and the most frequently used in Budapest (Szaller et al. 2014).

**Table 1:** Investigated tree species by sampling locations, and the nearest reference stations of Hungarian Air Quality Network (HAQN)

Tree species	Sampling locations			
	Buda arboretum	Krisztina street	Andrássy avenue	Karolina street
*Acer platanoides* L.’Globosum’	Acer Arbor	Acer Krisztina		
*Fraxinus excelsior* L ’Westhof’s Glorie’	Fraxinus Arbor		Fraxinus Andrássy	
*Tilia tomentosa* Moench	Tilia Arbor			Tilia Karolina
Nearest station of HAQN	PM Kosztolányi (Buda 1)	PM Széna (Buda 2)	PM Erzsébet (Pest)	PM Kosztolányi (Buda 1)

The first experimental site is located at the university campus in Buda. This area, the Buda Arboretum, is a 7.5-ha park under suburban conditions, on the south slope of Gellért-Hill, with low traffic around it; the oldest trees are about 120 years old. We used this site as a control site and collected samples from trees representing all the tree species mentioned above (Table 1). The second site is a small park with Norway maple (*Acer platanoides* L. ‘Globosum’) trees near Krisztina Street (Buda side), which is surrounded by heavy traffic on both sides. The nearest station of HAQN is at Széna Square (Buda 2). The third sampling site is a tree alley consisting of common ash (*Fraxinus excelsior* L. ‘Westhof’s Glorie’) species on Andrássy Avenue (Pest side). Here, the closest HAQN station is at Erzsébet Square (Pest). The fourth site is a tree alley consisting of silver linden (*Tilia tomentosa* Moench) trees, on the heavily trafficked Karolina Street, located in Buda. The nearest HAQN sampling station is on Kosztolányi Square (Buda 1).

According to the literature (and easily observed), the investigated tree species differ in leaf surface structure. Leaves of Norway maple are bright green, glabrous (no hairs) and lustrous beneath, bearded in the axis of the veins. Common ash also has glabrous leaves, though villous along the midrib beneath. In contrast, the leaves of silver lindens are slightly pubescent above and white tomentose beneath (Krüssmann 1976–1978; Rehder 1990; Trees and Shrubs Online 2017).

### Sampling, foliar dust and leaf analysis

Samples were collected at each site on the same days under rainless conditions. We collected and tested leaf samples in three periods from each site. In the first period, leaves were picked weekly on Thursdays, between May 13—May 27 in 2015 (three sampling days, 20th–22nd week of the year). The second sampling period was between October 30—November 12 in 2015 (three sampling days in 44th–46th week of the year). In the third sampling period, leaves were picked weakly between 5 and 19th October in 2016 (three sampling days in 40th–42nd week of the year).

Foliage was picked from the lower part of the canopy at an approximate height of 2–3 m. For each tree species, five trees of approximately the same age (between 15 and 25 years) were selected on each site for sampling, and six sample leaves were taken from each tree, randomly from all sides of the crown (30 fully developed sample leaves from each species and sites). Leaf samples were put carefully in paper bags and brought to the laboratory.

From each sample, the single leaf area of 10 leaves was measured, and then this was used to approximately calculate the average leaf area for each species and each location. The individual leaf areas were measured by leaf area meter AM 350 (ADC BioScientific Ltd, UK). Another 10 fresh leaves (subsamples) were washed and soaked in 250 ml distilled water for 20 h, and then a 10-min ultrasonic shaking was applied (Margitai and Braun 2005a, b; Margitai et al. 2005). Most researchers washed off the dust depositions from the leaves using deionized water (Kretinin and Selyanina 2006; Hofman et al. 2014; Chaudary and Rathore 2018), while Simon et al. (2011) combined water with 20-min shaking in plastic boxes. Antisari et al. (2012) confirmed Steubing (1982) that washing from 5 to 15 min considerably increases the yield but increasing the washing time to 30 min does not remove any more pollutants. Ataabadi et al. (2012) found a 10-min washing time to be most suitable.

The dust and dry particles-containing suspension was evaporated, and the weight of the residue was measured again and chemically investigated by using concentrated nitric acid–hydrogen peroxide. From the extract, five of the top ten (Hoodaji et al. 2012) air polluting HM (Pb, Fe, Ni, Cu and Zn) were determined by using the AURORA AI 1200 AAS appliance (AURORA 2005; Braun et al. 2007; Margitai and Braun 2005a, b; Margitai et al. 2005; Tandon 2013). The AAS equipment was operated with an air–acetylene flame and the built-in deuterium (D2) background correction was used. The precision of the AAS device is 0.5%. Detection limits: Pb: 0.1 mg dm^−3^, Fe: 0.03 mg dm^−3^, Ni: 0.07 mg dm^−3^, Zn: 0.01 mg dm^−3^, Cu: 0.02 mg dm^−3^ (AURORA 2005). The amount of the dust deposit washed from leaves and the HM content of this dust deposit were calculated to one m^2^ leaf area, weighted by the average leaf surface of the samples (mg m^–2^).

The leaf samples collected in the spring of 2015 and autumn of 2015 were dried to constant weight in an oven following washing. To analyze the leaf metal content, the ground dry leaf samples were digested at 105 °C with 10 ml concentrated nitric acid (HNO_3_) plus 4 ml 30% solution of H_2_O_2_, which was boiled until clearing. The contents of mineral nutrient elements in the two digestion solutions were measured using ICP atomic emission spectroscopy (ICP Thermo Jarrell Ash ICAP 61E equipment). The ICP was operated with 99.99% pure argon gas, and the dynamic background correction by spectrum shifter was used. Detection limits: Pb: 0.01 mg dm^−3^, Fe: 0.01 mg dm^−3^, Ni: 0.02 mg dm^−3^, Zn: 0.005 mg dm^−3^, Cu: 0.01 mg dm^−3^ (Jarvis et al. 1994; Winge et al. 1985). The detected HM content of the leaf samples after foliar dust removal was calculated in ration to the dry weight (mg kg^–1^) of the leaf samples.

### Data on precipitation and PM10 pollution in the air

For comparison with the foliar dust deposit data, the PM10 pollution data of the Hungarian Air Quality Network are presented in Figs.  2, 3 and 4. Although the foliar dust contains a wider range of particular matter sizes, we focused on the PM10 data as an indicator of the actual air pollution. This assumption matches Hofman et al. (2014) who found that in Antwerp (Belgium) 90% of water soluble dust deposit belongs to the > 10 μm fraction, while in oasis city Aksu (NW-China) Baidourela and Zhayimu (2015) found 93–97% for > PM10 fraction. We present the air pollution data starting four weeks prior to the first leaf sampling. The course PM10 concentration is presented weekly, means of the seven days prior to the sampling day are calculated.

 To compare the foliar dust deposit to the precipitation over the sampling periods in both years, the total rainfall during the seven days preceding each leaf collection is presented in Figs. 5 and 6. The precipitation data starting four weeks prior to the first leaf sampling are also included.

### Data analysis

In designing the sampling of the leaves, the principles of randomized complete block designs were followed: we consider the three seasons as main factors, the subplots were the species/location in six variants (Table 1), and the samples collected in three subsequent weeks were considered as replicates. The significance of each factor (seasons, species/location) was determined using analysis of variance (ANOVA) tests. After checking the normal distribution and homogeneity of variances, means of variables (dust deposit, Pb, Fe, Ni, Zn and Cu deposit on leaves and the leaf HM content) were separated by Tukey’s post hoc test (*p* < 0.05) using the IBM SPSS 20 software package.

## Results

### The dust and HM content washed off from the leaf surface

The statistical analysis indicates that the season significantly affects the foliar dust deposition (*F* = 23.351; Sig = 0.000), but the effect of tree species on different locations (*F* = 0.386; Sig = 0.855) was not significant (Table 2). The total dry residue weight of foliar dust deposition removed from the leaf samples during washing showed significant differences among the sampling seasons.

**Table 2:** Results of statistical analysis by ANOVA

Source	Sum of squares	df	Mean square	F	Sig
*Dependent variable: dust deposit of 3 sampling seasons*					
Corrected Model	472,313.278	17	27,783.134	2.923	0.003
Intercept	1,093,708.563	1	1,093,708.563	115.063	0.000
Season	443,919.263	2	221,959.632	23.351	0.000
species/location	18,350.525	5	3670.105	0.386	0.855
Season* spec/loc	10,043.490	10	1004.349	0.106	1.000
*Dependent variable: dust deposit of 2 sampling seasons*					
Corrected Model	15,117.602	11	1374.327	3.930	0.002
Intercept	268,626.279	1	268,626.279	768.152	0.000
Season	0.269	1	0.269	0.001	0.978
species/location	14,208.335	5	2841.667	8.126	0.000
Season* spec/loc	908.998	5	181.800	0.520	0.759
*Dependent variable: Pb deposit*					
Corrected Model	532.813	17	31.342	10.327	0.000
Intercept	1119.833	1	1119.833	368.976	0.000
Season	124.045	5	24.809	8.174	0.000
species/location	348.230	2	174.115	57.369	0.000
Season* spec/loc	60.539	10	6.054	1.995	0.063
*Dependent variable: Fe deposit*					
Corrected Model	483.621	17	28.448	7.651	0.000
Intercept	780.834	1	780.834	210.011	0.000
Season	96.899	5	19.380	5.212	0.001
species/location	336.342	2	168.171	45.231	0.000
Season* spec/loc	50.380	10	5.038	1.355	0.240
*Dependent variable: Ni deposit*					
Corrected Model	961.963	17	56.586	20.087	0.000
Intercept	1990.834	1	1990.834	706.695	0.000
Season	488.858	2	244.429	86.766	0.000
species/location	385.922	5	77.184	27.398	0.000
Season* spec/loc	87.184	10	8.718	3.095	0.006
*Dependent variable: Zn deposit*					
Corrected Model	108.346	17	6.373	6.942	0.000
Intercept	373.031	1	373.031	406.327	0.000
Season	29.727	2	14.863	16.190	0.000
species/location	68.921	5	13.784	15.015	0.000
Season* spec/loc	9.699	10	0.970	1.056	0.419
*Dependent variable: Cu deposit*					
Corrected Model	24.683	17	1.452	243.520	0.000
Intercept	35.236	1	35.236	5909.781	0.000
Season	15.094	2	7.547	1265.788	0.000
species/location	7.288	5	1.458	244.480	0.000
Season* spec/loc	2.301	10	0.230	38.586	0.000

**Fig. 1:**
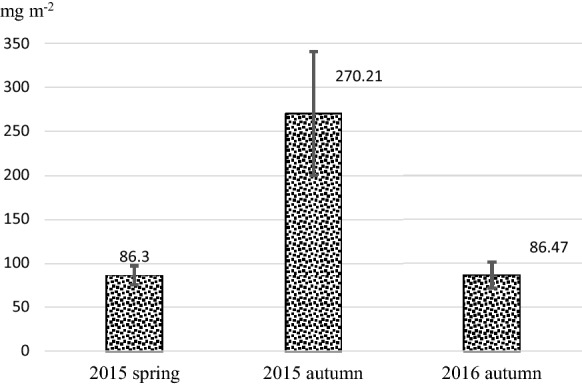
Dust deposition washed off from leaves in Budapest (mg m^–2^): average of all samples (means are separated by Tukey's test)

On leaf samples collected in spring 2015 (19th–25th weeks) and autumn 2016 (40th–42nd weeks), we measured 86.3 mg m^–2^ and 86.5 mg m^–2^ foliar dust (average of the six species/location samples). However, on 2015 autumn samples (44th–46th weeks of the year) the average dust deposit was 270.2 mg m^–2^, more than three times higher than that of the two other sampling periods (Fig. 1).

As the high foliar dust deposit measured in the autumn of 2015 may have masked the effect of species/location, the data of the other two seasons (2015 spring and 2016 autumn) were examined separately (Table 2, dust deposit of 2 sampling seasons). The analysis showed a significant effect of the species/location factor (*F* = 8.126; Sig = 0.000; Table 2). On the leaves of silver linden, collected in Karolina Street, in both the spring of 2015 and autumn of 2016, we measured the highest leaf dust deposit (116.90 mg m^–2^ and 130.0 mg m^–2^, respectively), while the lowest dust deposit was measured on maple trees 66.5 mg m^–2^ (2015) and on common ash 58.6 mg m^–2^ (2016) (Table 3).

**Table 3:** Dust deposition washed off from leaves of different species in Budapest (mg m^–2^): average of samples from spring 2015 to autumn 2016

Sampling	Acer Arbor		Acer Krisztina		Fraxinus Arbor		Fraxinus Andrássy		Tilia Arbor		Tilia Karolina		Mean
Spring 2015	66.5	ab	88.3	abc	67.1	ab	76.5	ab	102.5	bcd	116.9	bcd	86.3
Autumn 2016	81.8	ab	81.3	ab	58.6	a	72.6	ab	94.5	abc	130.0	d	86.5
Mean	74.2	a	84.8	ab	62.8	a	74.6	a	98.5	bc	123.5	c	

Means were separated by Tukey’s test, and values followed by the same letters within rows are not significantly different at *p* = 0.5

The statistical analysis of the investigated factors indicates that the sampling seasons and the sampled tree species across different locations significantly affect the content certain elements (we focused on Pb, Fe, Ni, Zn and Cu). The F values and analysis of significance for seasonal effects on Pb content (*F* = 8.174 Sig = 0.000), on Fe content (*F* = 5.212; Sig = 0.000), on Ni content (*F* = 86.766; Sig = 0.000), on Zn content (*F* = 16.19; Sig = 0.000) and on Cu content (*F* = 1265.788; Sig = 0.000) show that the HM content of dust deposit significantly differs by investigated seasons. Similarly, the F values and analysis of significance for species/location on Pb content (*F* = 57.369; Sig = 0.000), on Fe content (*F* = 45.231; Sig = 0.000), on Ni content (*F* = 27.398; Sig = 0.000), on Zn content (*F* = 15.015; Sig = 0.000) and on Cu content (*F* = 244.480; Sig = 0.000) show significantly different HM content of dust deposit by species/location (Table 2).

From among the investigated HM elements, Pb (7.23 mg m^−2^ and 5.29 mg m^−2^) and Fe (6.3 mg m^−2^ and 4.71 mg m^−2^) content in foliar dust deposit was significantly higher in both autumnal sampling periods compared to the spring of 2015 (Table 4). The Ni content of dust deposit was the highest in the spring of 2015 (9.44 mg m^−2^) followed by the autumn of 2016 (6.63 mg m^−2^) and the autumn 2015 (2.14 mg m^−2^) with significant differences. The Cu showed significantly higher deposition in samples from the spring of 2015 (1.54 mg m^−2^) compared to the other two seasons (Table 4), while Zn deposit was significantly higher in the two sampling seasons in 2015 (3.49 mg m^−2^ and 2.71 mg m^−2^).

**Table 4:** HM deposition washed off from leaves in Budapest (mg m^−2^) in three sampling seasons (spring 2015, autumn 2015 and 2016)

Sampling season	Pb deposit		Fe deposit		Ni deposit		Zn deposit		Cu deposit	
Spring 2015	1.14	a	0.40	a	9.44	c	3.49	b	1.54	b
Autumn 2015	7.23	b	6.30	c	2.14	a	2.71	b	0.58	a
Autumn 2016	5.29	b	4.71	b	6.63	b	1.68	a	0.31	a

Means were separated by Tukey’s test, and values followed by the same letters within columns do not differ significantly at *p* = 0.5

The HM content (of the five investigated elements Pb, Fe, Ni, Zn and Cu) detected in the dry residue of dust deposition on leaf samples (mg m^–2^ leaf) on urban trees in Budapest differed significantly by tree species/location (Table 5). The highest HM content was found in each sampling period on silver linden leaves collected from the heavily trafficked Karolina Street (Tilia/Karolina), followed by the leaves of same species collected from the Buda Arboretum (Tilia/Arbor). Leaves of Norway maple and common ash collected from Buda Arboretum showed the lowest HM content, but samples of the same species collected from locations with similarly busy traffic showed intermediate values in Fe (Andrássy Street) and Pb and Cu content on leaves of Norway maple collected from Krisztina Street (Acer/Krisztina). For each species, the samples collected from street location compared to those collected from Buda Arboretum did not show statistically significant differences, albeit the tendency is visible that street locations contained higher amounts of HM deposits.

**Table 5:** HM deposition washed off from leaves in Budapest (mg m^−2^) of different species sampled in different locations

Species/location	Acer Arbor		Acer Krisztina		Fraxinus Arbor		Fraxinus Andrássy		Tilia Arbor		Tilia Karolina	
Pb deposit	3.33	a	4.34	ab	3.21	a	3.20	a	6.45	ab	6.79	b
Fe deposit	2.15	a	4.17	ab	2.37	a	3.54	ab	4.50	ab	6.10	b
Ni deposit	4.11	a	5.60	a	3.59	a	3.67	a	9.4	b	10.16	b
Zn deposit	1.68	a	2.44	a	1.56	a	1.8	a	3.75	b	4.54	b
Cu deposit	0.55	a	0.82	ab	0.40	a	0.50	a	1.22	b	1.36	b

Means were separated by Tukey’s test, and values followed by the same letters within rows do not differ significantly at *p* = 0.5

### The HM content detected in the leaves of urban trees after removal by washing off the dust deposition

After washing off the dust deposition from the leaves, the heavy metal content of the dried leaf samples showed differences between May and November in 2015 (Table 6). The statistical analysis implied significant effects of season on Pb, Fe, Zn.

**Table 6:** Leaf tissue HM content of urban trees in Budapest at spring 2015 and autumn 2015

Tree species	*Acer platanoides* ‘Globosum’				*Fraxinus excelsior* ‘Westhof’s Glorie’				*Tilia tomentosa*			
Date	Spring 2015		Autumn 2015		Spring 2015		Autumn 2015		Spring 2015		Autumn 2015	
Pb mg kg^–1^	0.80	a	1.69	b	0.89	a	1.54	b	0.88	a	2.11	c
Fe mg kg ^–1^	147.00	ab	248.00	c	112.00	a	218.00	bc	127.00	a	285.00	c
Ni mg kg^–1^	1.17	a	0.92	a	1.97	b	1.06	a	1.30	ab	1.30	ab
Zn mg kg^–1^	32.60	b	32.30	b	30.60	b	22.70	a	19.30	a	20.85	a
Cu mg kg^–1^	13.03	a	14.70	a	11.56	a	12.90	a	26.10	a	15.40	a

Means were separated by Tukey’s test, and values followed by the same letters within rows are not significantly different at* p* = 0.5

Considering the two sampling dates, the Fe and Pb contents of the leaves were significantly higher when collected in early November. The Fe and Pb content of the leaves of the three investigated tree species did not show significant differences among samples collected at the same time. The Cu and Ni contents of the leaves were not significantly different from spring to autumn, nor were there statistically significant difference among the three tree species.

The Zn content in the Norway maple (Table 6) was the highest in both sampling dates, but the leaves of the common ash collected in the spring of 2015 contained significantly higher level of Zn (30.6 mg kg^–1^) compared to those collected in the autumn (22.70 mg kg^–1^).

## Discussion

**Fig. 2:**
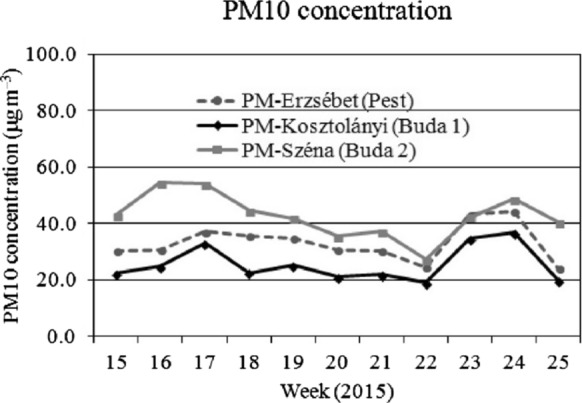
Weekly average concentration of PM10 (in 3 measuring sites) from 15 to 25th week of 2015 (µg m^–3^). Source of data: Hungarian Air Quality Network

**Fig. 3:**
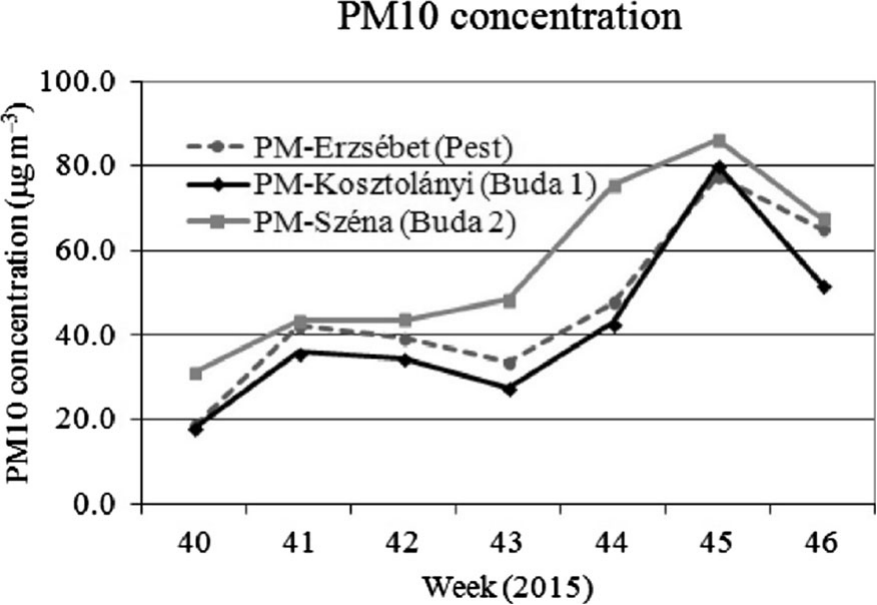
Weekly average concentration of PM10 (in 3 measuring sites) from 40 to 46th week of 2015 (µg m^3^). Source of data: Hungarian Air Quality Network

**Fig. 4:**
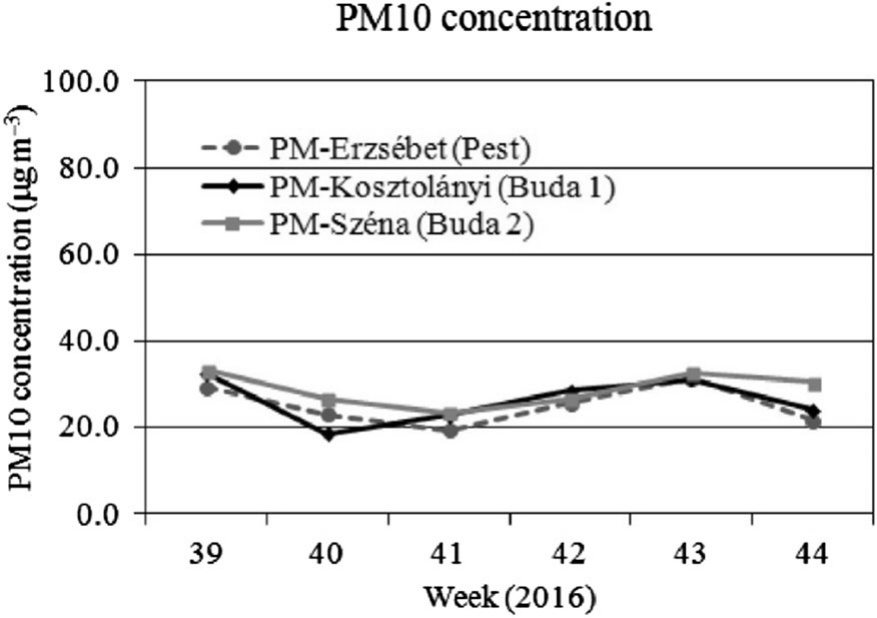
Weekly average concentration of PM10 (in 3 measuring sites) from 39 to 44th week of 2016. Source of data: Hungarian Meteorological Service

We found significant differences in average foliar dust deposition between the three sampling periods (Fig.  1). The dust deposition in the spring of 2015 (20th–22nd weeks) and in the autumn of 2016 (44th–46th weeks) was 86.3 mg m^–2^ and 86.5 mg m^–2^, respectively, while in the autumn of 2015 (40th–42nd weeks) the weight of foliar dust was about 3 times higher (270.2 mg m^–2^, Table 3). Comparing our data to the PM10 concentration in the air measured in locations close to our sampling sites (Figs.  2, 3 and 4, data from HAQN), we conclude that the extremely high foliar dust deposition in the fall of 2015 reflects the high PM10 concentration in the air at that time. In the spring of 2015, the PM10 concentration in the air was between 19–54 µg m^–3^, while in the fall of that year it was between 18–33 µg m^–3^, and in the autumn of 2015 in the 46th weeks the weekly mean PM10 concentration increased to 86 µg m^–3^, due to a longer cold period, which increased the intensity of communal heating. Hofman et al. (2014) found that in Antwerp (Belgium) 90% of water soluble dust deposit belongs to the > 10 μm fraction, while in oasis city Aksu (NW-China) Baidourela and Zhayimu (2015) found 93–97% for > PM10 fraction. Although the foliar dust contains both smaller and larger size fractions of PM than PM10, our results support the opinion of researchers (Beckett et al. 2000b; Mori et al. 2015; Sæbø et al. 2012 and Simon et al. 2014) that the foliar dust deposit of urban trees is good indicator of air pollution.

**Fig. 5:**
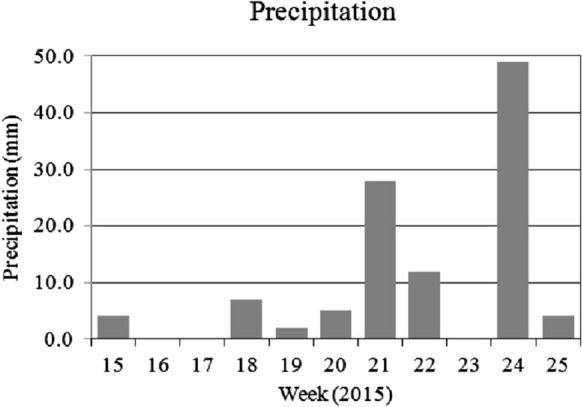
Weekly precipitation in spring sampling weeks of 2015. Source of data: Hungarian Meteorological Service

The amount of rainfall may also have contributed to the amount of foliar dust measured. Nowak et al. (2006) and Mori et al. (2015) showed that rain removes the pollutants from the leaf surface influencing the deposits of air pollutants on leaves of different species. According to the data of the Hungarian Meteorology Service, the monthly rainfall during the three sampling periods was fairly similar: in May and June 2015, there was an average amount of rain (Fig.  5), in October 2016 a low amount of rainfall was registered, but in both sampling periods the foliar dust deposit was low (Fig.  1).

**Fig. 6:**
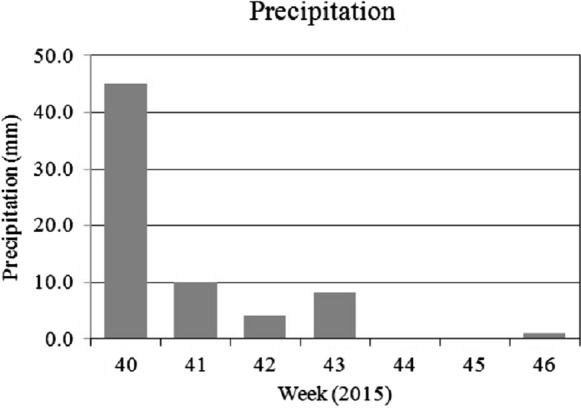
Weekly precipitation in autumn sampling weeks of 2015

In contrast, the highest dust deposition was measured in October 2015, when the monthly total rainfall was high (110 mm), albeit the sampling weeks (44th–46th) were almost rainless (1.1 mm total within 3 weeks, Fig.  6). When comparing the weekly precipitation data to the size of the foliar dust deposits in the sampling periods (20th–22nd weeks in 2015 and 44th–46th week in 2015), we can detect the influence of rainfall on the foliar dust deposits (Fig.  6).

The foliar dust deposit on leaves collected from the same tree species in Buda Arboretum (7.5 ha garden in suburban conditions) and in locations in Budapest with heavy automobile traffic did not differ significantly (Table 3). Our results are in correspondence with Simon et al. (2014), who also did not find significant differences in dust deposition on leaves between urban and rural conditions. This underlines the fact that the leaf surface of trees along heavily trafficed streets and in large parks provide similar dust removal capacity.

Our data on foliar dust deposits also agree with the literature reporting large differences between tree species (Beckett et al. 2000a, b; Kretinin and Selyanina 2006; Yuan et al. 2009; Sæbø et al. 2012; Simon et al. 2014; Mori et al. 2015; Chen et al. 2016). Data from the autumn of 2015 indicate a saturation of dust deposits on the leaves, which might result in no differences between species. Sæbø et al. (2012) found about 60–700 mg m^–2^ PM deposits depending on the species: for *Acer platanoides* low, around 100 mg m^–2^, for *Fraxinus excelsior* 80–130 mg m^–2^. They evaluated *Acer platanoides* and *Fraxinus excelsior* as less efficient in trapping of PM from air pollution, which is confirmed by our results. In contrast Kretinin and Selyanina (2006) classified the dust retaining capacity of the Norway maple as low (7 mg m^–2^), on the common ash moderate (41.5 mg m^–2^), which are much lower capacities than that amount of foliar dust measured in our samples. Supposedly the above authors evaluated the tree species under less polluted conditions. Simon et al. (2014) reported 20–150 mg m^–2^ leaf dust for the *Padus serotina*, *Acer campestre* and *Quercus robur* with glabrous leaves while the leaves of *Celtis occidentalis* and *Acer negundo* with trichomas collected around 400 mg m^–2^ leaf dust. In correspondence with above results, the pubescent leaf surface (Krüssmann 1976–1978; Rehder 1990) of silver linden results in higher dust adsorbent capacity that we observed in the Karolina Street samples. Although there are no data on the dust retaining capacity of silver linden, Kretinin and Selyanina (2006) reported very high capacities for similar species (*Tilia* × *europaea* and *Tilia platyphyllos*), 464.6 mg m^–2^ and 313.6 mg m^–2^, respectively. These data are close to our results (270.2 mg m^–2^) measured in the autumn of 2015, when the air PM10 concentration was very high. Our results from those sampling periods with low PM10 concentration in the air (2015 May–June and 2016 October) underline the high dust entrapping capacity of silver linden (*Tilia tomentosa*), which are the first quantified data for this taxa (Table 3). The high dust entrapping efficiency of silver linden leaves is mostly likely due to its hairy leaves, although further leaf characteristics like stomatal structure (Mori et al. 2015) need further investigations. Drought and heat tolerance (Tóth et al. 2015) make this species suitable for urban forestry.

The deposit of investigated HM elements showed temporal differences (Table 4): in spring of 2015, the deposit of Ni, Cu was significantly higher than that of both autumn samples, while Zn was higher in both 2015 samples compared to 2016. This suggests that the deposit of HM elements in air pollution may change temporarily, partly due to their variable concentration in air, which should be considered when HM content of foliar dust is applied in biomonitoring (Hoodaji et al. 2012). Another reason for the decreasing rate of Zn, Cu and Ni could be that these elements occur in compounds that might be easily washed off by rain or removed by wind (Mori et al. 2015). In contrast, Pb and Fe deposits were significantly higher in both autumn samples compared to the spring of 2015, which indicates the accumulative character of both HM elements in dust deposition.

Comparing the capacity of the leaves to adsorb HM particles across the investigated tree species, we can state that the most efficient species was the silver linden followed by the Norway maple trees and then the common ash in each sampling periods (Table 5). Our results confirm Nowak et al. (2006); Simon et al. (2011, 2014); Mori et al. (2015) who reported large differences between tree species in HM retaining capacity in foliar dust. The large dust and HM removal capacity of tree foliage, according to Yuan et al. (2009), can provide health related benefits in urban settings.

Low deposition of investigated HM-s was found on leaves from the common ash and the Norway maple without any significant differences between the two locations (Table 4). The Pb deposit on the common ash leaves in the spring of 2015 was at a similar level to that found by Aksoy and Demirezen (2006) in Turkey. However, on autumn leaves both in 2015 and 2016, the deposits in Budapest were much higher. The significantly higher Fe, Pb, Zn, Cu, Ni deposition on silver linden leaves emphasizes the larger HM adsorbing capacity of linden leaves. For both Norway maple and silver linden, we could not find data on HM deposition in the literature, so our research provides this first quantified data (Table 5).

Comparing the HM deposit of leaf samples collected in spring and autumn of 2015 (Table 4), there is a conspicuous change between the seasons. The Fe and Pb deposits on leaves increased until the end of the season when it reached 5–15 times higher levels, while the weight of the Zn, Cu and Ni deposit decreased. This performance of Pb and Fe deposits indicates an accumulating character, in contrast to the other investigated HMs. This might be caused, on the one hand, by the presence of different concentrations of the HMs in air pollution, or perhaps by an unknown interaction between the leaves and the HM particles. We leave this question to future work.

The investigation of the leaf samples after the removal of the surface dust (Table 6) showed that Zn, Cu and Ni contents were typical to tree species without any significant differences between the seasons and locations, while Fe and Pb showed seasonal differences. Compared to the data of Uhrin and Supuka (2018), we found on Norway maple leaves similar amounts of Fe content in the spring and higher amounts in autumn, lower Pb content in both samples, while the Zn and Cu contents were more than twice as much. Hrotkó et al. (2014b) found leaf Fe concentration in cherry (*Prunus avium* L.) similarly to our May leaf samples, but the autumn samples of urban trees contained higher levels. Our results partly corresponds to Guha et al. (1966) and Kim and Fergusson (1994) who found that concentration of Fe, Ni, and Pb in leaves shows a steady rise until senescence, while the concentration of Zn and Cu essential elements falls gradually until senescence. In leaves from the Norway maple and the common ash, the Fe content increased by 60% over the season, while in silver linden the Fe content approximately doubled. Similarly, the Pb content of leaves of all the three species doubled by the autumn. The increased Fe and Pb contents of autumnal leaves confirm Hoodaji et al. (2012) too, who supposed that a proportion of heavy metals is taken up by plant, but Zn and Cu compounds might be better leached by surface and subterranean runoff.

Except in regard to Ni concentrations, our results for all three species are in correspondence to the recent results of Tomašević and Aničić (2010) and Popović et al. (2010) and confirm the obvious increase of Pb and Fe concentrations from May to September. Popović et al. (2010) and Tomašević et al. (2011) found that the seasonal accumulation of the examined elements in leaves of *Aesculus hippocastanum* L. was more regular. Aznar et al. (2009) supposed that Pb, as a nonessential metal, remained in the foliage as part of a detoxification process. Hovmand et al. (2009) studied the origin of Pb in Norway spruce and Tomašević and Aničić (2010) the origin of Pb in horse chestnut tree leaves. They showed that less than 2% of the Pb content comes from root uptake, and approximately 98% is of the atmospheric origin. These findings support our statement that the increased Fe and Pb content in our leaf samples might be originated mainly from leaf surface, although the root uptake (Piczak et al. 2003) cannot be excluded. Based on these results, we can conclude that Pb and Fe accumulate in leaves from May to November and the source of accumulation can be the Pb and Fe deposits on the leaves.

Our results partly confirm Popović et al. (2010) and Tomašević et al. (2011) who suggested the leaf Pb content for monitoring of atmospheric Pb pollution, but we emphasize the importance of autumnal sampling. On the other hand, for biomonitoring of Ni, Zn and Cu air pollution the HM detection in leaf samples is not recommended. Considering our results on the HM content of foliar dust and the leaf HM content, we can conclude that the HM deposits in foliar dust on Norway maple and silver linden trees seems to be efficient in monitoring HM air pollution for Fe, Pb, Zn, Cu and Ni. Although Aksoy and Demirezen (2006) suggested using common ash for biomonitoring, due to the low removal capacity of its leaves, our recommendation would be to rather use Norway maples or silver lindens.

In summary, our data on leaf dust deposits are valuable and provide new insights into the environmental services of major urban tree. Our research results proved our hypothesis (1) and (2): that the investigated trees species showed different dust and HM entrapping capacity of leaves, but the differences between tree species might be eliminated under extremely high air pollution. Our research provides the first quantitative data on dust and HM deposit on leaves of *Tilia tomentosa*, which proved to be very efficient both in foliar dust and HM retaining capacity. Furthermore, we confirmed previous studies on dust deposit on *Acer platanoides* and *Fraxinus excelsior* leaves, and we provide the first data on the HM capturing capacity of their leaves. Our data indicate a specific performance of Pb and Fe deposits on the leaves, which accumulates throughout the growing season, in contrast to other heavy metals. This specific performance should be considered when foliar dust deposits are used in monitoring air pollution. Our research results could not prove hypothesis (3) completely. The leaf Fe and Pb contents after the removal of the foliar dust indicate that the leaves absorb these heavy metals from deposits on the leaf surface; thus, the leaf Fe and Pb contents are a suitable indicator of Fe and Pb air pollution.

## Conclusion

Foliar dust data may contribute to the estimation of the dust removal capacity of urban trees. The dust deposition on leaves of urban trees reflected the dust pollution in the air; thus, foliar dust is a suitable indicator for monitoring dust and HM pollution in the air.

Urban trees differ in dust removal capacity: the highest performer in this regard was the silver linden, significantly lower were the Norway maple and the common ash. Silver lindens with their pubescent (hairy) leaf surface proved to be the most efficient in entrapping and retaining dust and heavy metals.

The investigated HM elements within these deposits showed seasonal differences: Pb and Fe deposits were the highest in autumn samples, while Ni, Zn and Cu were the highest in the spring samples. By the end of the season, the Fe and Pb contents of the deposits on the leaf surface increased to five to ten times higher levels, while the other HMs did not show such accumulation.

The increased Fe and Pb contents of autumn leaf samples after the foliar dust was washed off indicate that over the course of the season the leaves absorb Fe and Pb from the dust deposit. Thus, the Fe and Pb contents of leaf tissue are a suitable indicator of air Fe and Pb pollution, but this does not hold for Ni, Zn and Cu pollution.
